# Metabolic dysfunction-associated steatotic liver disease, metabolic alcohol-related liver disease, and incident dementia: a nationwide cohort study: MASLD, MetALD, and dementia risk

**DOI:** 10.1186/s12876-025-03814-1

**Published:** 2025-04-29

**Authors:** Woo-Young Shin, Eun Seok Kang, Yun Hwan Oh, Meng Sha, Qiang Xia, Seogsong Jeong, Yoosun Cho

**Affiliations:** 1 Department of Family Medicine, Chung-Ang University Gwangmyeong Hospital, Chung-Ang University College of Medicine, 110 Deokan-Ro, Gwangmyeong-Si, Gyeonggi-Do South Korea; 2 Department of Medicine, College of Medicine, Chung-Ang University, Seoul, Republic of Korea; 3 Department of Biomedical Informatics, Korea University College of Medicine, 73 Goryeodae-Ro, Seongbuk-Gu, Seoul, 02841 Republic of Korea; 4 Department of Liver Surgery, School of Medicine, Renji Hospital, Shanghai Jiao Tong University, Shanghai, China

**Keywords:** Metabolic dysfunction-associated steatotic liver disease, Metabolic alcohol-associated liver disease, Fatty liver index, Dementia, Aged

## Abstract

**Background:**

The relationship between the newly proposed steatotic liver disease (SLD) subtypes—metabolic dysfunction-associated steatotic liver disease (MASLD) and metabolic alcohol-associated liver disease (MetALD)—and dementia is understudied. We evaluated the dementia risk associated with these subtypes.

**Methods:**

This retrospective cohort study included 296,001 participants aged over 60 who underwent health examinations between 2009 and 2010. Participants were categorized into non-SLD (reference), MASLD, and MetALD groups and followed up until dementia onset, death, or December 31, 2019. SLD was defined by a fatty liver index ≥ 30, with (i) MASLD based on cardiometabolic risk factors, and (ii) MetALD as MASLD with moderate alcohol intake. Outcomes included overall dementia, Alzheimer's disease (AD), and vascular dementia (VaD). Subdistribution hazard ratios (SHRs) was calculated using the Fine–Gray model, treating death as a competing risk.

**Results:**

Over 7,430,253 person-years of follow-up, 11,345 dementia cases occurred (10,863 AD and 2,159 VaD). Adjusted SHRs for MASLD were 1.10 (1.07–1.13) for AD and 1.20 (1.13–1.27) for VaD. For MetALD, SHRs were 0.90 (0.87–0.94) for AD and 1.53 (1.40–1.66) for VaD. Dementia risk in both MASLD and MetALD increased over longer periods, with MetALD initially linked to increased VaD risk and decreased AD risk, which reversed after three years.

**Conclusions:**

MASLD and MetALD were associated with increased risks of AD and VaD; MetALD showing a stronger association with VaD. Understanding the distinct effects of different SLD subtypes on dementia is crucial for improving risk assessment and management strategies.

**Supplementary Information:**

The online version contains supplementary material available at 10.1186/s12876-025-03814-1.

## Introduction

Metabolic dysfunction-associated steatotic liver disease (MASLD), recently renamed from nonalcoholic fatty liver disease (NAFLD) to better reflect its cardiometabolic associations, is now the most prevalent chronic liver disease, affecting over 30% of the global adult population [1]. MASLD often coexists with obesity and abnormal glucose and lipid metabolism [2], and is a known risk factor for hypertension, diabetes, and cardiovascular/cerebrovascular diseases [3–6]. Recent evidence suggests a link between MASLD and cognitive impairment, including dementia, sharing common risk factors such as insulin resistance, obesity, hypertension, and dyslipidemia [7]. Growing evidence indicates a direct connection between MASLD and structural brain changes that may contribute to dementia development driven by the following mechanisms: increased brain insulin resistance leading to oxidative stress, excessive free fatty acids, and mitochondrial dysfunction [7, 8]; and liver fat-induced inflammation activating microglial cells and increasing inflammatory cytokine expression in the brain [9].

However, current studies demonstrated no association between NAFLD and cognitive impairment or risk of dementia; supported by two promising studies, the Rotterdam study [10] and UK Biobank data [11]. The Rotterdam Study, with 5.7-year of follow up period, found no association between NAFLD and cognitive impairment or dementia risk in older adults, and even suggested a potential early protective effect of NAFLD against dementia [10]. Similarly, a meta-analysis of UK Biobank participants over a 12.4-year follow-up period found no significant association between NAFLD and the risks of all-cause dementia, Alzheimer's disease (AD) and vascular dementia (VaD) [11]. Despite these findings, it's important to note the limitations of these studies, such as their focus on European populations, relatively short follow-up periods in some cases, and heterogeneity in dementia definitions. While the UK Biobank meta-analysis included a Korean study showing increased dementia risk with NAFLD, it was predominantly weighted by European data. These limitations highlight the need for more long-term studies examining Asian-specific dementia risk associated with steatosis liver disease (SLD).With dementia projected to affect over 152 million people globally by 2050 [12], and the new criteria for SLD and its sub-classifications, including MASLD, MASLD with increased alcohol intake (MetALD), and alcoholic liver disease (ALD) [13], have been proposed, evidence-based validation in real cohorts is crucial to clarify these associations.

Most recently, a 13-year follow-up cohort study of 403,506 middle-aged and older participants from the UK Biobank used a newly proposed sub-classification of SLD that encompasses alcohol consumption along with cardiometabolic risk factors and evaluated in relation to dementia [14]. The study reported that both MASLD and MetALD were associated with an increased risk of VaD, but showed no association or even a reduced risk of AD [14], which is the opposite result as compared to our previous study that found a higher risk of AD in NAFLD patients [15]. Therefore, there is insufficient evidence regarding the association between SLD subtypes and the risk of AD or VaD, especially for MetALD.

While some evidence suggests a potential link between MASLD/MetALD and dementia, more research is needed to understand this relationship, particularly in Asian populations. Asian populations exhibit distinct characteristics in obesity and metabolic health compared to Western populations. The higher prevalence of fatty liver in lean individuals in Asia, higher body fat percentages at lower BMI levels [16], is associated with cardiometabolic complications [17, 18] and increased mortality [19], potentially influencing disease progression and dementia risk [14]. The higher burden of viral hepatitis [16], although excluded from our study, could potentially interact with MASLD/MetALD and affect cognitive outcomes [20, 21]. Given these implications, it is crucial to explore aspects of the Asian context that are not captured in Western-centric studies. Therefore, in this large cohort of elderly Koreans, we aimed to investigate the association of MASLD and MetALD with the development of dementia, including AD, and VaD, compared to those without SLD.

## Materials and methods

### Study population

**Fig. 1:**
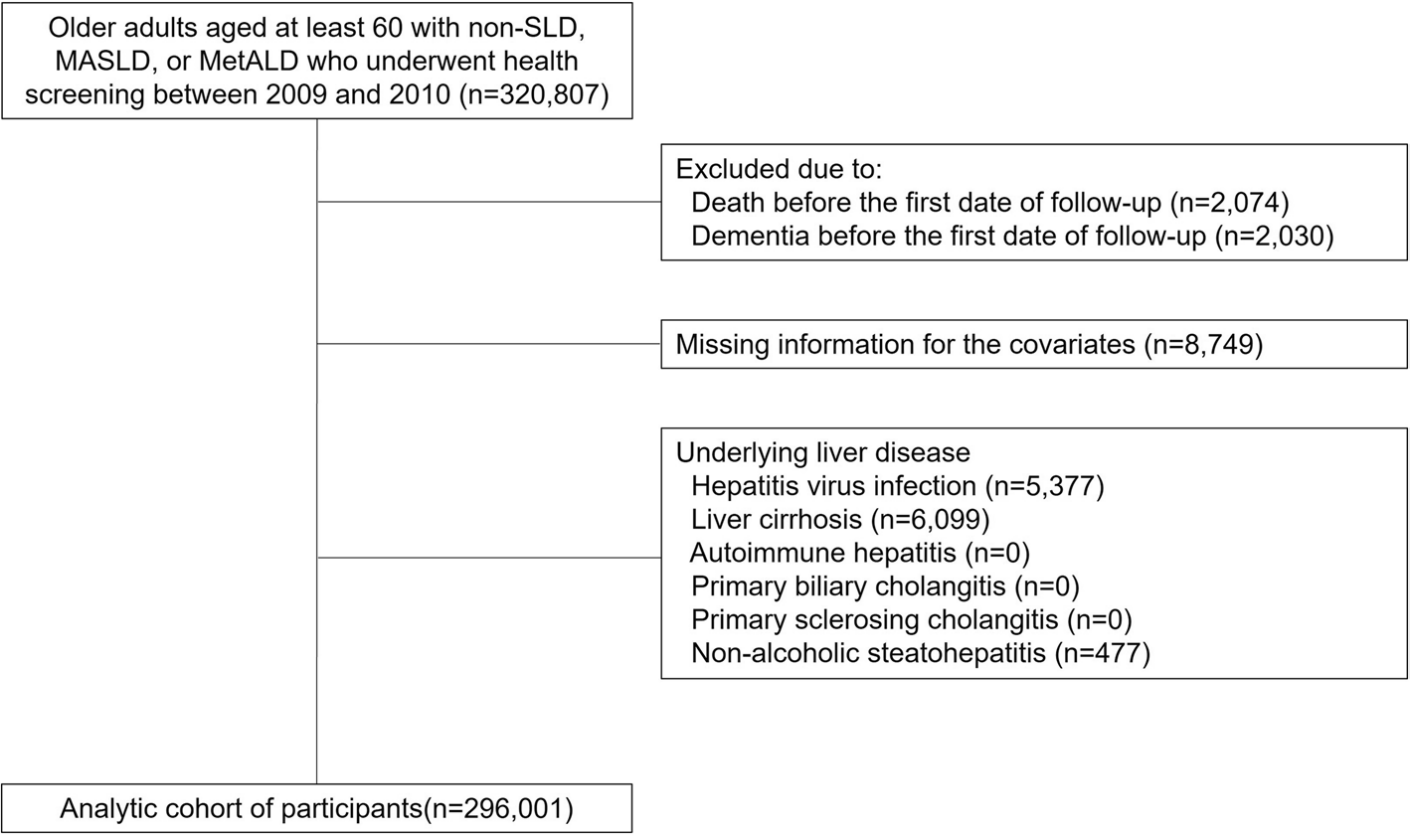
Flow diagram for the inclusion of the older adults. Study participants with non-SLD (without any cardiometabolic risk factors), MASLD, and MetALD defined in the health screening examination between 2009 and 2010 were derived from the National Health Insurance Service-Senior Cohort after excluding participants with death, history of dementia, missing information for the covariates, and underlying liver diseases

The dataset for this study was sourced from the NHIS-Senior cohort V2.0, a hybrid cohort of administrative data, prospectively and retrospectively gathered. The National Health Insurance Service (NHIS) of Korea ensures universal health coverage for its citizens, providing complete medical services with a participation rate of approximately 97% [22]. It systematically gathers extensive data, including socio-demographic profiles, medication prescriptions, medical histories, and records from hospital and outpatient visits. Additionally, the NHIS conducts comprehensive biennial health screenings, collecting data on laboratory tests, lifestyle questionnaires, and body measurements. This cohort includes 511,953 individuals aged 60 and older, randomly selected from a pool of 6.4 million seniors in 2008 (NHIS-2024-11-2-054) [23]. Our study analysed data collected from 2009 to 2019, focusing on 320,807 seniors screened for non-SLD, MASLD, or MetALD from 2009 to 2010. Exclusions were made for participants who passed away before the follow-up period (*n* = 2,074), had dementia (*n* = 2,030), lacked necessary covariate data (*n* = 8,749), or a history of other liver diseases, including viral hepatitis infection, non-alcoholic steatohepatitis, and liver cirrhosis (*n* = 11,953; Fig. 1). Ultimately, the study included 296,001 elderly participants. The Chung-ng University Gwangmyung Hospital’s Institutional Review Board approved this study (No.: 2307–097 - 072), and the requirement for informed consent was omitted due to the anonymized and confidential .

### Diagnosis of non-SLD, MASLD, and MetALD

SLD was determined using the fatty liver index (FLI), setting a cut-of point of 30 or higher. The FLI was calculated using the following formula [24]:

$$\text{FLI}=({\text{e}}^{0.953\times \text{loge}(\text{triglycerides}) +0.139\times \text{body mass index}+0.718\times \text{loge}(\upgamma -\text{glutamyl transferase})+0.053\times \text{waist circumference}-15.745)}/({1}^{+\text{e}0.953\times \text{loge}(\text{triglycerides})+0.139\times \text{body mass index}+ 0.718\times \text{loge}(\upgamma -\text{glutamyl transferase})+0.053\times \text{waist circumference}-15.745})\times 100.$$

FLI is a non-invasive surrogate for hepatic steatosis, showing an area under the receiver operating characteristic curve of 0.844 [25]. It has positive predictive values of 83.2% for males and 84.8% for females, and negative predictive values of 65.3% for males and 87.4% for females in Asian populations [25].

MASLD is diagnosed through the detection of hepatic steatosis and one or more of the following five cardiometabolic risk factors: (i) a body mass index (BMI) of at least 23 kg/m^2^ or a waist measurement exceeding 90 cm for males and 85 cm for females [26]; (ii) blood sugar level of 100 mg/dL or above, or documented adult-onset diabetes or its treatment; (iii) blood pressure at or above 130/85 mmHg, or treatment with antihypertensive medications; (iv) triglyceride levels at or above 150 mg/dL, or the use of lipid-lowering treatments; (v) HDL cholesterol under 40 mg/dL for males and under 50 mg/dL for females, or undergoing lipid-lowering therapy. MetALD was defined as MASLD coinciding with moderate alcohol intake, defined as 30 to 60 g daily for males and 20 to 50 g daily for females [13].

### Diagnosis of overall dementia, AD and VaD

Overall dementia was identified using International Classification of Diseases, Tenth Revision (ICD- 10) codes: F00, F01, F02, F03, and G30, supported by the prescription of medications typically associated with dementia treatment, such as donepezil, galantamine, rivastigmine, and memantine. AD was diagnosed using ICD- 10 codes F00 and G30, while VaD was determined using ICD- 10 code F01, contingent on the use of aforementioned dementia-related medications. We monitored all study participants from their initial health screening until the occurrence of dementia, death, or December 31 st, 2019. The follow-up investigation was carried out respectively for AD and VaD.

### Statistical analysis

Continuous variables were reported as means with standard deviations for normally distributed data, or medians with interquartile ranges for data that were not normally distributed. Group differences were assessed using the independent t-test for normally distributed variables and the Mann–Whitney U test for non-normally distributed variables. Categorical variables were expressed as counts and percentages, with group differences evaluated using the chi-squared test. We employed Cox proportional hazards regression to compute cause-specific adjusted hazard ratios (aHRs) with 95% confidence intervals (CIs). The initial model adjusted minimally for age (as a continuous variable in years), sex (as a categorical variable; male or female), and BMI (as a continuous variable in kg/m^2^). A more comprehensive adjustment model included these variables plus the Charlson comorbidity index (CCI) (as categories: 0, 1, and ≥ 2), smoking status (categorized as never, past, and current), and levels of moderate-to-vigorous physical activity (MVPA) (categorized as none, 1–2 times/week, 3–4 times/week, and ≥ 5 times/week). The covariates were selected based on previous studies that examined their effects on the risk of dementia [27–31]. Data on alcohol consumption was collected from the self-reported Alcohol Use Disorder Identification Test questionnaire. The Fine-Gray competing risk model adjusted for multiple variables was utilized to estimate the subdistribution hazard ratio (SHR), which indicates the probability of developing dementia in the presence of competing risks of death, in assessing the risk associated with different subtypes of SLD. The assumption of proportional hazards was verified using the Kolmogorov-type supremum test. Dementia incidence was calculated as the number of events per 1,000 person-years.

Inverse probability of treatment weighting (IPTW) was applied and variance inflation factors were computed. To reduce heterogeneity between the non-SLD versus MASLD and non-SLD versus MetALD groups, inverse probability of treatment weighting (IPTW) was applied using age, sex, BMI, household income, CCI [32], smoking status, and MVPA as covariates. Variance inflation factors were computed to assess the collinearity among the covariates. To validate the robustness of the results, sensitivity analyses and subgroup analyses were performed. In sensitivity analyses, we excluded dementia cases within the initial one, two, and three years of follow-up to eliminate pre-existing cases. Subgroup analysis investigated the diversity in dementia risk associated to MASLD and MetALD subtypes. Stratification was based on sex, body mass index (BMI), smoking status, moderate-to-vigorous physical activity (MVPA), Charlson Comorbidity Index (CCI), and cardiometabolic conditions such as hypertension, type 2 diabetes, and dyslipidemia. Data collection, mining, and statistical analyses were conducted using SAS Enterprise Guide (version 8.3, SAS Institute, Cary, NC, USA).

## Results

### Baseline characteristics of each subtype of SLD

Table 1 presents the baseline characteristics of the study participants in the Korean NHIS-Senior cohort. Study participants included 129,580 (43.8%), 153,992 (52.0%), and 12,429 (4.2%) participants with non-SLD, MASLD, and MetALD, respectively. A higher proportion of males was evident in the MetALD group (93.5%) compared to the MASLD (48.4%) and non-SLD (34.8%) groups. Average ages were 68.3 years for non-SLD, 67.6 years for MASLD, and 66.4 years for MetALD, indicating a younger demographic in the MetALD group.

**Table 1:** Descriptive characteristics of the study population in the National Health Insurance Service-Senior Cohort across the subtypes of SLD

Characteristic	Non-SLD (*n* = 129,580)	MASLD (*n* = 153,992)	MetALD (*n* = 12,429)
Age, years	68.3 (5.8)	67.6 (5.5)	66.4 (5.1)
Sex, n (%)			
Male	45,100 (34.8)	74,466 (48.4)	11,621 (93.5)
Female	84,480 (65.2)	79,526 (51.6)	808 (6.5)
Household income^a^, n (%)			
1 st quartile (lowest)	17,525 (13.5)	20,371 (13.2)	1,453 (11.7)
2nd quartile	21,412 (16.5)	27,195 (17.7)	2,599 (20.9)
3^4 d^ quartile	31,702 (24.5)	39,161 (25.4)	3,438 (27.7)
4 th quartile (highest)	58,941 (45.5)	67,265 (43.7)	4,939 (39.7)
Body mass index, kg/m^2^	22.2 (2.4)	25.7 (2.7)	24.7 (2.7)
Waist circumference, cm	77.7 (6.5)	87.2 (7.0)	87.4 (7.1)
Systolic blood pressure, mmHg	126.2 (15.9)	131.0 (15.7)	133.3 (16.0)
Diastolic blood pressure, mmHg	76.6 (9.9)	79.3 (9.9)	80.9 (10.0)
Fasting serum glucose, mg/dL	98.8 (23.2)	106.7 (29.7)	109.8 (30.1)
Total cholesterol, mg/dL	195.8 (37.0)	202.9 (40.1)	195.5(37.5)
HDL-cholesterol, mg/dL	56.3 (27.2)	52.5 (28.2)	56.4 (29.1)
LDL-cholesterol, mg/dL	118.9 (35.7)	119.3 (40.3)	106.4 (38.3)
Triglycerides, mg/dL	107.9 (52.5)	163.6 (88.2)	170.5 (101.8)
Alanine aminotransferase, IU/L	18.6 (8.8)	27.1 (19.0)	28.6 (20.2)
Aspartate aminotransferase, IU/L	23.7 (7.8)	27.6 (15.0)	32.1 (22.3)
γ-GT, IU/L	17.6 (7.6)	30 (22–44)	53 (35–88)
Alcohol consumption, n (%)			
No	106,482 (82.2)	113,211 (73.5)	0 (0)
1–2 times/week	15,665 (12.1)	29,913 (19.4)	910 (7.3)
3–4 times/week	4,573 (3.5)	8,035 (5.2)	5,905 (47.5)
≥ 5 times/week	2,860 (2.2)	2,833 (1.9)	5,614 (45.2)
Cigarette smoking, n (%)			
Never	101,306 (78.2)	107,955 (70.1)	3,866 (31.1)
Past	14,419 (11.1)	26,199 (17.0)	4,054 (32.6)
Current	13,855 (10.7)	19,838 (12.9)	4,509 (36.3)
MVPA, n (%)			
No	90,948 (70.2)	106,569 (69.2)	7,779 (62.6)
1–2 times/week	12,854 (9.9)	15,837 (10.3)	1,376 (11.1)
3–4 times/week	10,383 (8.0)	12,588 (8.2)	1,232 (9.9)
≥ 5 times/week	15,395 (11.9)	18,998 (12.3)	2,042 (16.4)
Charlson comorbidity index, n (%)			
0	40,190 (31.0)	40,757 (26.5)	4,153 (33.4)
1	40,140 (31.0)	46,427 (30.2)	4,080 (32.8)
≥ 2	49,250 (38.0)	66,808 (43.4)	4,196 (33.8)

Continuous data are presented as mean (standard deviation) and median (interquartile range) if normally distributed and not normally distributed, respectively

Categorical data are expressed as the number (%)

*Acronyms*: *SLD* steatotic liver disease, *MASLD* metabolic dysfunction-associated steatotic liver disease, *MetALD* metabolic dysfunction-associated steatotic liver disease with increased alcohol intake, *HDL* high-density lipoprotein, *LDL* low-density lipoprotein cholesterol, *γ-GT* γ-glutamyl transpeptidase, *MVPA* moderate-to-vigorous physical activity

^a^Proxy for socioeconomic status based on the insurance premium of the National Health Insurance Service

### Risk of incident dementia across SLD subtypes before the IPTW

Over 7,430,253 person-years of follow-up, 11,345 dementia cases occurred (10,863 AD and 2,159 VaD). The association between SLD subtypes and incident dementia before implementing IPTW is depicted (Supplemental Table 1, 2, and 3). Compared to individuals with non-SLD, aHRs with 95% CI among those with MASLD showed a significant increased risk of overall dementia, AD, and VaD after fully adjustment of confounders including age, sex, BMI, household income, CCI, smoking status, and MVPA. The participants with MetALD had a significantly increased risk of incident VaD, with an aHR of 1.46 (95% CI, 1.15–1.85) in the fully adjusted model (Supplemental Table 1). Using Fine-Gray regression model, which accounts for overall death as a competing event (Supplemental Table 2 and 3), the presence of MetALD was significantly associated with an increased risk of VaD only after adjusting confounders.

### Risk of incident dementia across SLD subtypes after the IPTW

After weighting, standardized mean differences between non-SLD and MASLD groups were all < 0.10 in absolute value, indicating balanced covariates (Supplemental Table 4). Comparing non-SLD to MetALD, there was less covariate balance, but standardized mean differences improved after IPTW weighting. We presented the descriptive characteristics of participants with non-SLD and MASLD, as well as those with non-SLD and MetALD (Supplemental Table 5 and 6).

In the non-SLD versus MASLD comparison, we observed 283,319 and 286,134 participants, respectively (Table 2). Compared to the participants with non-SLD, the SHR (95% CI) for incident overall dementia among those with MASLD was 1.11 (1.08–1.14) after adjusting potential confounders including age, sex, body mass index, household income, CCI, smoking, and MVPA. In the non-SLD versus MetALD comparison (140,995 non-SLD and 201,374 MetALD), the SHR (95% CI) for incident overall dementia among those with MetALD was 0.99 (0.95–1.03) after adjusting confounders (Table 2). Furthermore, we evaluated the risk of incident specific types of dementia, AD and VaD, according to the presence of MASLD and MetALD. The SHR (95% CI) for incident AD among those with MASLD was 1.10 (1.07–1.13) after adjusting confounders, in contrast, the SHR (95% CI) for incident AD among those with MetALD was 0.90 (0.87—0.94). For the risk of VaD, the SHRs (95% CI) among those with MASLD and MetALD were 1.20 (1.13–1.27) and 1.53 (1.40–1.66), respectively. The variance inflation factors for the variables used in evaluating these SHRs were within acceptable limits, ensuring no undue inflation of the regression coefficients (Supplemental Table 7).

**Table 2:** SHRs for incident dementia across SLD subtypes after the inverse probability of treatment weighting

	Non-SLD	MASLD	*P* value	Non-SLD	MetALD	*P* value
Participant, n	281,319	286,134		140,995	201,374	
Competing event, n	33,150	44,337		18,607	73,078	
**Overall dementia**						
Event, n	10,513	11,758		5,577	7,621	
PYs	2,359,475	2,346,445		1,171,649	1,444,080	
Incidence/1,000 PYs	4.46	5.01		4.76	5.28	
SHR (95% CI)^a^	1.00 (reference)	1.11 (1.09–1.14)	< 0.001	1.00 (reference)	0.95 (0.91–0.98)	0.005
SHR (95% CI)^b^	1.00 (reference)	1.11 (1.08–1.14)	< 0.001	1.00 (reference)	0.99 (0.95–1.03)	0.652
**Alzheimer’s disease**						
Event, n	10,109	11,253		5,377	6,942	
PYs	2,363,327	2,351,067		1,173,657	1,446,008	
Incidence/1,000 PYs	4.28	4.79		4.58	4.80	
SHR (95% CI)^a^	1.00 (reference)	1.11 (1.08–1.14)	< 0.001	1.00 (reference)	0.88 (0.85–0.92)	< 0.001
SHR (95% CI)^b^	1.00 (reference)	1.10 (1.07–1.13)	< 0.001	1.00 (reference)	0.90 (0.87–0.94)	< 0.001
**Vascular dementia**						
Event, n	1,956	2,388		1,026	1,853	
PYs	2,388,519	2,377,442		1,187,101	1,453,398	
Incidence/1,000 PYs	0.82	1.00		0.86	1.27	
SHR (95% CI)^a^	1.00 (reference)	1.21 (1.14–1.29)	< 0.001	1.00 (reference)	1.36 (1.25–1.47)	< 0.001
SHR (95% CI)^b^	1.00 (reference)	1.20 (1.13–1.27)	< 0.001	1.00 (reference)	1.53 (1.40–1.66)	< 0.001

SHRs (95% CIs) were calculated using the Fine-Gray regression with overall death as a competing event

*Acronyms*: *SHR* subdistribution hazard ratio, *SLD* steatotic liver disease, *MASLD* metabolic dysfunction-associated steatotic liver disease, *MetALD* metabolic dysfunction-associated steatotic liver disease with increased alcohol intake, *IPTW* inverse probability of treatment weighting, *PY* person-year, *CI* confidence interval

^a^Adjusted for age, sex, and body mass index

^b^Adjusted for age, sex, body mass index, household income, Charlson comorbidity index, smoking, and moderate-to-vigorous physical activity

### Sensitivity analyses for incident overall dementia across the SLD subtypes after the IPTW

Additionally, we evaluated the risk of incident overall dementia, AD, and VaD across SLD subtypes after IPTW, considering different latent periods (Table 3). The risk of incident overall dementia persistently increased in those with MASLD and MetALD with increasing latent periods. Interestingly, MetALD was initially associated with a decreased risk of AD in the first two years, but after three years, the MetALD-related AD risk significantly increased. For incident VaD risk, MetALD status was strongly associated with an increased risk of VaD development with an increasing latent period, reaching an SHR of 1.73 (95% CI 1.57–1.90) at three years. The increased risk of MASLD-related AD or VaD remained significant as the latent period increased. When BMI was excluded from the adjustment variables, both MASLD and MetALD were associated with a higher risk of VaD, but not for AD (Supplementary Table 8).

**Table 3:** Sensitivity analyses on SHRs for incident overall dementia across the SLD subtypes after the inverse probability of treatment weighting

Latent period	Non-SLD (*n* = 281,319)	MASLD (*n* = 286,134)	*P* value	Non-SLD (*n* = 140,995)	MetALD (*n* = 201,374)	*P* value
**Overall dementia**						
1-year						
SHR (95% CI)^a^	1.00 (reference)	1.11 (1.08–1.14)	< 0.001	1.00 (reference)	1.01 (0.97–1.05)	0.673
SHR (95% CI)^b^	1.00 (reference)	1.10 (1.07–1.13)	< 0.001	1.00 (reference)	1.06 (1.02–1.10)	0.005
2-year						
SHR (95% CI)^a^	1.00 (reference)	1.11 (1.08–1.15)	< 0.001	1.00 (reference)	1.06 (1.02–1.11)	0.004
SHR (95% CI)^b^	1.00 (reference)	1.11 (1.08–1.14)	< 0.001	1.00 (reference)	1.11 (1.07–1.16)	< 0.001
3-year						
SHR (95% CI)^a^	1.00 (reference)	1.11 (1.08–1.14)	< 0.001	1.00 (reference)	1.19 (1.14–1.24)	< 0.001
SHR (95% CI)^b^	1.00 (reference)	1.10 (1.07–1.14)	< 0.001	1.00 (reference)	1.25 (1.20–1.31)	< 0.001
**Alzheimer’s disease**						
1-year						
SHR (95% CI)^a^	1.00 (reference)	1.11 (1.08–1.14)	< 0.001	1.00 (reference)	0.92 (0.88–0.96)	< 0.001
SHR (95% CI)^b^	1.00 (reference)	1.11 (1.08–1.14)	< 0.001	1.00 (reference)	0.95 (0.91–0.98)	0.006
2-year						
SHR (95% CI)^a^	1.00 (reference)	1.12 (1.09–1.15)	< 0.001	1.00 (reference)	0.95 (0.91–0.99)	0.021
SHR (95% CI)^b^	1.00 (reference)	1.11 (1.08–1.14)	< 0.001	1.00 (reference)	0.98 (0.94–1.02)	0.250
3-year						
SHR (95% CI)^a^	1.00 (reference)	1.12 (1.09–1.15)	< 0.001	1.00 (reference)	1.06 (1.01–1.10)	0.013
SHR (95% CI)^b^	1.00 (reference)	1.11 (1.08–1.15)	< 0.001	1.00 (reference)	1.08 (1.04–1.13)	< 0.001
**Vascular dementia**						
1-year						
SHR (95% CI)^a^	1.00 (reference)	1.18 (1.11–1.26)	< 0.001	1.00 (reference)	1.44 (1.32–1.56)	< 0.001
SHR (95% CI)^b^	1.00 (reference)	1.17 (1.10–1.25)	< 0.001	1.00 (reference)	1.62 (1.48–1.77)	< 0.001
2-year						
SHR (95% CI)^a^	1.00 (reference)	1.15 (1.08–1.22)	< 0.001	1.00 (reference)	1.44 (1.32–1.58)	< 0.001
SHR (95% CI)^b^	1.00 (reference)	1.13 (1.06–1.21)	< 0.001	1.00 (reference)	1.61 (1.47–1.76)	< 0.001
3-year						
SHR (95% CI)^a^	1.00 (reference)	1.10 (1.03–1.17)	0.008	1.00 (reference)	1.55 (1.42–1.70)	< 0.001
SHR (95% CI)^b^	1.00 (reference)	1.08 (1.01–1.16)	0.023	1.00 (reference)	1.73 (1.57–1.90)	< 0.001

SHRs (95% CIs) were calculated using the Fine-Gray regression after excluding dementia events that occurred within specified periods

*Acronyms:* *SHR* subdistribution hazard ratio, *SLD* steatotic liver disease, *MASLD* metabolic dysfunction-associated steatotic liver disease, *MetALD* metabolic dysfunction-associated steatotic liver disease with increased alcohol intake, *CI* confidence interval

^a^Adjusted for age, sex, and body mass index

^b^Adjusted for age, sex, body mass index, household income, Charlson comorbidity index, smoking, and moderate-to-vigorous physical activity

### Subgroup analysis on the risk of incident overall dementia of MASLD after the IPTW

**Fig. 2:**
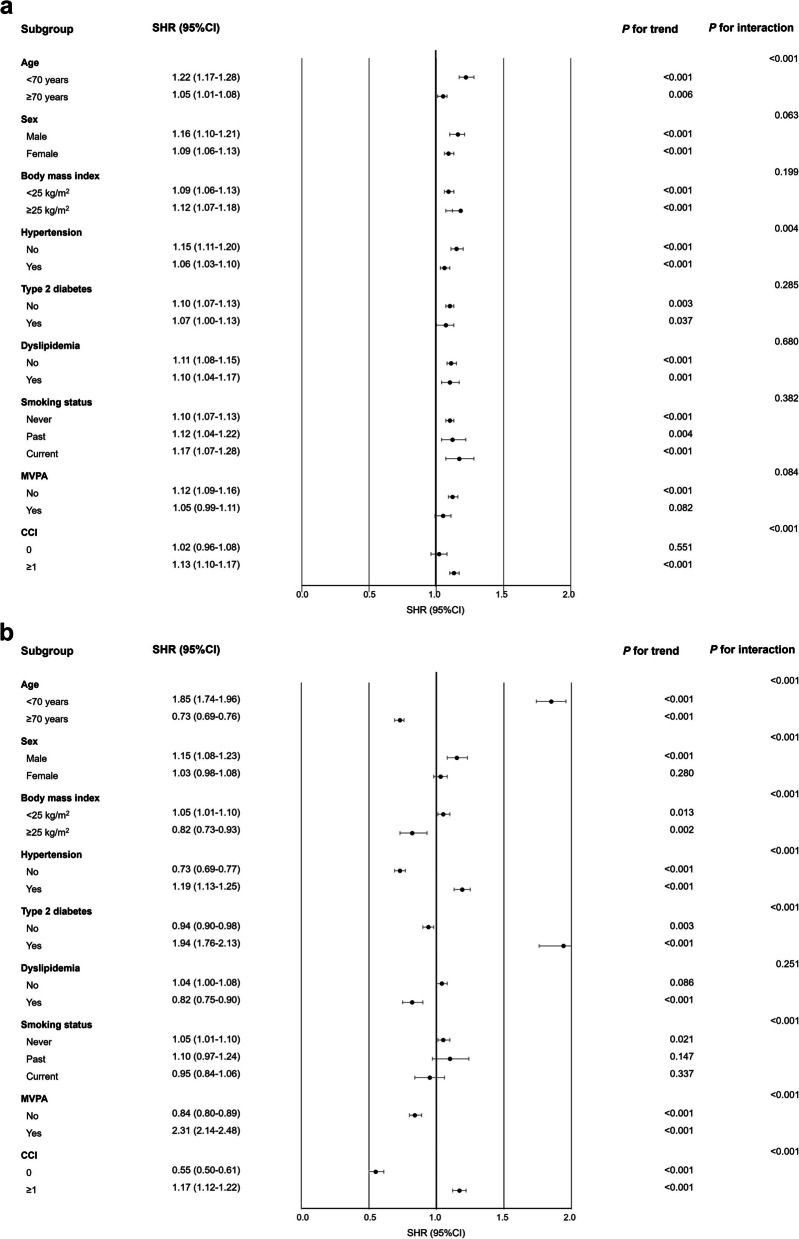
Stratified analyses on association of the SLD subtypes with the risk of overall dementia. Effects of MASLD versus non-SLD on the risk of dementia (Panel **A**) and MetALD versus non-SLD (Panel **B**) on the risk of dementia were evaluated using the Fine-Gray regression with overall death as competing risks after adjustments for age, sex, body mass index, household income, Charlson comorbidity index, smoking, moderate-to-vigorous physical activity after the inverse probability of treatment weighting

Additionally, we performed subgroup analysis to verify the heterogeneity in the risk of dementia associated with different SLD subtypes, MASLD and MetALD. Irrespective of sex, BMI, and cardiometabolic diseases including hypertension, type 2 diabetes, and dyslipidemia, participants with MASLD showed an increased risk of dementia. Younger participants (< 70 years), those who were physically less active, and those with more comorbidities tended to have a significantly higher risk of overall dementia compared to those without these conditions (Fig. 2a). Participants with MetALD who were younger (< 70 years), male, non-obese, physically more active, and had hypertension, type 2 diabetes, and comorbidities showed an increased risk of developing overall dementia. In contrast, those without hypertension, type 2 diabetes, and comorbidities exhibited a decreased risk of dementia (Fig. 2b).

## Discussion

In this large-scale cohort study, older adults with MASLD showed an increased risk of incident overall dementia, AD, and VaD. Conversely, those with MetALD showed no significant association with overall incident dementia; instead, they had an increased risk of VaD but a decreased risk of AD. Despite the initial short-term reduction in AD risk in MetALD, both MASLD and MetALD were associated with increased risks of AD and VaD after a 3-year of latent period, with MetALD showing a stronger association with VaD. These findings suggest that hepatic steatosis, cardiometabolic risk factors, and alcohol consumption defining MASLD and MetALD may additively influence the heterogeneous outcomes in the risk of dementia subtypes.

Our study is consistent with a previous epidemiological study for approximately 6 million Korean adults aged 40 years or older demonstrated a positive association between NAFLD and developing AD [15] and a matched cohort study of Swedish patients aged 65 years or older with NAFLD showed increased risk of overall dementia [33]. Emerging research has connected insulin resistance to various neurodegenerative processes in AD through pathways involving oxidative stress, mitochondrial dysfunction, and chronic liver inflammation, all contributing to neurodegeneration and cerebrovascular damage, which impact brain metabolism and lead to cognitive decline [7]. Therefore, we hypothesize that chronic low-grade hepatic inflammation may have attributed to the increased risk of dementia. Further studies are needed to identify whether modification of cardiometabolic risk factors and chronic liver inflammation attenuate dementia risk.

Given the potential risk of vascular damage driven by MASLD, individuals with MetALD might have a higher risk of VaD development, suggesting an additive effect of alcohol and cardiometabolic factors. In MetALD, alcohol interacts synergistically with metabolic risk factors to accelerate liver fibrosis [34] and promote endothelial dysfunction, thereby increasing the risk of vascular disease [35]. Alcohol consumption itself promotes these effects by inducing inflammation and oxidative stress [36–38]. Additionally, the'neurotoxicity'hypothesis proposes that chronic alcohol exposure directly induces neuronal damage through multiple mechanisms, including glutamate excitotoxicity, increased oxidative stress, and impaired neurogenesis [39].

Interestingly, MetALD initially appeared to reduce the risk of AD, but this risk increased after a 3-year latent period. To address potential survival bias and reverse causation, particularly among older participants who might represent healthier individuals less susceptible to metabolic syndrome [40], we incorporated a latent period in our analysis. This approach acknowledges the complex nature of MetALD, which involves both metabolic dysregulation and moderate alcohol consumption. Initially, individuals with MetALD may appear healthier, leading to a temporarily lower AD risk. However, persistent MetALD likely increases AD risk over time. Previous studies suggest that apparent associations between metabolic factors and dementia risk may be due to reverse causation bias, with prodromal dementia causing weight loss, lower blood pressure, and changes in serum lipid levels years before diagnosis [41, 42]. While observational studies have indicated that moderate alcohol consumption might protect against dementia [43, 44], a Mendelian randomization study reveals this as likely due to reverse causation and residual confounding rather than a true protective effect [45]. VaD often results from acute vascular events causing sudden cognitive declines, whereas AD pathology accumulates gradually, influenced by metabolic dysfunction. These factors, implicated in metabolic syndrome, may predispose individuals to future cognitive decline. Therefore, incorporating a latent period and excluding individuals with potential preclinical dementia may better reveal the association between MetALD and increased AD risk.

In our study, both MASLD and MetALD were significant risk factors for dementia, with a stronger association in participants under 70. Though attenuated in those aged 70 and older, the risk remained significant for MASLD, while MetALD was associated with a decreased dementia risk compared to non-SLD individuals in this age group. Previous studies have shown an increased risk of cognitive dysfunction and incident dementia in middle-aged adults with NAFLD [46], which did not persist in older adults without dementia by age 70 [47]. This could suggest selection bias, as severe MASLD cases in middle-aged individuals might lead to their exclusion from the study, resulting in an older cohort with milder MASLD. Furthermore, the impact of metabolic dysfunction on dementia development might be more significant in younger age groups due to reduced metabolic flexibility, leading to heightened inflammatory responses, oxidative stress, and a stronger immune response [48].

While MASLD was associated with incident dementia regardless of BMI, MetALD was associated with incident dementia only in non-obese individuals. This is consistent with the Framingham Offspring study, which found midlife obesity to increase dementia risk, whereas obesity at older ages (70 years and above) was associated with lower risk after accounting for competing mortality risks [49]. The increased risk of dementia in non-obese individuals with MetALD might be due to reverse causation; obesity can be a consequence rather than a cause of better nutrition or less severe dementia, as weight loss, considered a manifestation of dementia, may precede its onset of dementia [50]. The"obesity paradox"suggests that higher BMI in older adults is associated with lower mortality and, in some cases, a reduced risk of dementia, providing a metabolic buffer against aging and the neurodegenerative process [49, 51]. However, the protective effect of higher BMI in older adults may be limited if accompanied by sarcopenic obesity, the co-occurrence of sarcopenia and obesity, has been associated with increased dementia risk [52]. A large-scale study found that sarcopenic obesity was associated with higher dementia risk [52]. This suggests that the combination of excess fat and muscle loss may be particularly detrimental to cognitive health. Changes in body composition, rather than BMI alone, appear to be crucial in determining dementia risk. A recent study involving over 13 million adults found that increases in muscle mass were associated with decreased dementia risk, while increases in body fat significantly increased dementia risk [53]. Specifically, when body fat increased by 1 kg/m^2^, the risk of dementia increased by 19% for men and 53% for women [53]. Further research is needed to elucidate the mechanisms behind the notable association between SLD and dementia in the non-obese population, focusing on detailed body composition and obesity phenotypes.

There are inherent limitations in this study. First, SLD was operationally defined using the FLI. Although further validation using radiologic or pathologic methods may enhance the association between SLD and dementia, the FLI evaluation offers the advantage of identifying individuals who have not been diagnosed by a physician into MASLD [54], potentially resulting in a higher proportion of individuals in the MASLD group. The prevalence of MASLD was higher for males aged between 50 and 69 years, whereas it was higher in females aged between 65 to 79 years with an age-standardized FLI-defined MASLD prevalence of 51.7% in 2010 to 2011 among Korean adults [55]. Secondly, the absence of data on individuals'lifetime drinking habits raises with a concern that former drinkers or those who ceased alcohol consumption for health reasons possibly being categorized as non-drinkers, and since the data is self-reported, recall bias could affect the reliability of the findings. Thirdly, this study did not consider genetic or biological factors related to SLD-associated dementia. For instance, APOE polymorphisms are significantly associated with non-alcoholic steatohepatitis, particularly the a APOE ε3 genotype. The APOE ε4 isoform increases the risk for neurodegenerative diseases [56]. The PNPLA3 rs738409 G allele predisposes to fatty liver while modestly protecting against coronary artery disease [56]. It is also associated with greater cerebral white matter hyperintensity and microbleeds [57]. These findings highlight the intricate relationships between genetic factors, liver disease, and vascular health, which may collectively influence dementia risk, emphasizing the need for further comprehensive research in this area. Fourthly, individuals with non-alcoholic steatohepatitis, a progressed SLD with a significant inflammation and cell damage, were excluded from the analyses to determine the effects of MASLD, a systemic form of hepatic steatosis that involves hepatic fat accumulation and low-grade chronic liver inflammation, on the risk of dementia. Future studies that evaluate the effects of metabolic dysfunction-associated steatohepatitis on dementia risk is warranted.

In conclusion, the newly proposed SLD highlights that individuals with MASLD and MetALD are at an increased risk of developing dementia in the older Asian population. Specifically, MASLD was associated with increased risk of incident dementia including AD and VaD. MetALD was associated with increased risk of VaD and eventually AD with extended latent periods, despite initially reducing the risk of AD. Our findings suggest that identifying patients with SLD based on hepatic steatosis, metabolic complexity, and alcohol consumption could clarify their association with the risk of incident dementia and its subtypes, potentially offering distinct strategies to mitigate the development of dementia in both MASLD and MetALD groups.

## Supplementary Information

Supplementary Material 1.

## Data Availability

The datasets generated during and/or analyzed during the current study are available from a third party (National Health Insurance Service, https://nhiss.nhis.or.kr). Researchers can write a research proposal to the National Health Insurance Service to get the data after an approval.

## Abbreviations

AD: Alzheimer’s disease
CI: Confidence interval
FLI: Fatty liver index
HR: Adjusted hazard ratios
MASLD: Metabolic dysfunction-associated steatotic liver disease
MetALD: Metabolic alcohol-associated liver disease
MVPA: Moderate-to-vigorous physical activity
NAFLD: Nonalcoholic fatty liver disease
SD: Standard deviation
SHR: Subdistribution hazard ratio
SLD: Steatotic liver disease
VaD: Vascular dementia
