# Supplementary material for: Metabolic dysfunction-associated steatotic liver disease, metabolic alcohol-related liver disease, and incident dementia: a nationwide cohort study: MASLD, MetALD, and dementia risk
Source: BMC Gastroenterol. 2025 Apr 29;25:308. doi: 10.1186/s12876-025-03814-1 (PMC12039214; doi:10.1186/s12876-025-03814-1)
Supplement: Supplementary file 1 — Supplementary Material 1. [file 12876_2025_3814_MOESM1_ESM.docx]

**Supplemental Tables**

**Supplemental Table 1.** Cause-specific aHRs for incident dementia across SLD subtypes.

**Supplemental Table 2.** SHRs for incident overall dementia across SLD subtypes.

**Supplemental Table 3.** SHRs for incident Alzheimer’s disease and vascular dementia across SLD subtypes.

**Supplemental Table 4.** Standardized mean differences of variables before and after the IPTW.

**Supplemental Table 5.** Descriptive characteristics of the participants with non-SLD and MASLD after the inverse probability of treatment weighting.

**Supplemental Table 6.** Descriptive characteristics of the participants with non-SLD and MetALD after the inverse probability of treatment weighting.

**Supplemental Table 7.** Variance inflation factors for variables used in evaluating subdistribution hazard ratios for incident overall dementia after the inverse probability of treatment weighting across the SLD subtypes.

**Supplemental Table 8.** Subdistribution hazard ratios for incident overall dementia and its subtypes across SLD subtypes after excluding body mass index from the adjustment.

**Supplemental Table 1.** **Cause-specific aHRs for incident dementia across SLD subtypes.**

| **Outcome** | **aHR (95% CI)**^†^ | ***P* value** | **aHR (95% CI)**^‡^ | ***P* value** |
| --- | --- | --- | --- | --- |
| Overall dementia |  |  |  |  |
| No SLD | 1.00 (reference) |  | 1.00 (reference) |  |
| MASLD | 1.12 (1.07-1.17) | <0.001 | 1.09 (1.04-1.15) | <0.001 |
| MetALD | 1.11 (0.99-1.25) | 0.084 | 1.09 (0.97-1.29) | 0.147 |
| Alzheimer’s disease |  |  |  |  |
| No SLD | 1.00 (reference) |  | 1.00 (reference) |  |
| MASLD | 1.11 (1.06-1.17) | <0.001 | 1.09 (1.04-1.14) | <0.001 |
| MetALD | 1.08 (0.96-1.22) | 0.209 | 1.06 (0.94-1.20) | 0.326 |
| Vascular dementia |  |  |  |  |
| No SLD | 1.00 (reference) |  | 1.00 (reference) |  |
| MASLD | 1.17 (1.05-1.31) | 0.005 | 1.13 (1.01-1.26) | 0.029 |
| MetALD | 1.53 (1.21-1.93) | <0.001 | 1.46 (1.15-1.85) | 0.006 |

HRs (95% CIs) were calculated using the Cox proportional hazards regression.

^†^Adjusted for age, sex, and body mass index.

^‡^Adjusted for age, sex, body mass index, household income, Charlson comorbidity index, smoking, and moderate-to-vigorous physical activity.

Acronyms: aHR, adjusted hazard ratio; CI, confidence interval; SLD, steatotic liver disease; MASLD, metabolic dysfunction-associated steatotic liver disease; MetALD, metabolic dysfunction-associated steatotic liver disease with increased alcohol intake.

**Supplemental Table 2. SHRs for incident overall dementia across SLD subtypes.**

|  | **Non-SLD** | **MASLD** | ***P* value** | **MetALD** | ***P* value** |
| --- | --- | --- | --- | --- | --- |
| Participants, n | 129,580 | 153,992 |  | 12,429 |  |
| Event, n | 5,294 | 5,726 |  | 325 |  |
| PYs | 1,075,700 | 1,285,989 |  | 103,223 |  |
| SHR (95% CI)^†^ | 1.00 (reference) | 1.09 (1.04-1.15) | <0.001 | 1.09 (0.97-1.22) | 0.160 |
| SHR (95% CI)^‡^ | 1.00 (reference) | 1.07 (1.02-1.12) | 0.004 | 1.08 (0.96-1.21) | 0.205 |

SHRs (95% CIs) were calculated using the Fine-Gray regression with overall death as a competing event.

^†^Adjusted for age, sex, and body mass index.

^‡^Adjusted for age, sex, body mass index, household income, Charlson comorbidity index, smoking, and moderate-to-vigorous physical activity.

Acronyms: SHR, subdistribution hazard ratio; SLD, steatotic liver disease; MASLD, metabolic dysfunction-associated steatotic liver disease; MetALD, metabolic dysfunction-associated steatotic liver disease with increased alcohol intake.

**Supplemental Table 3. SHRs for incident Alzheimer’s disease and vascular dementia across SLD subtypes.**

|  | **No SLD (n=129,580)** | **MASLD (n=153,992)** | ***P* value** | **MetALD (n=12,429)** | ***P* value** |
| --- | --- | --- | --- | --- | --- |
| Alzheimer’s disease |  |  |  |  |  |
| Event, n | 5,102 | 5,459 |  | 302 |  |
| PYs | 1,077,613 | 1,288,407 |  | 103,376 |  |
| SHR (95% CI)^†^ | 1.00 (reference) | 1.08 (1.03-1.14) | 0.001 | 1.06 (0.94-1.20) | 0.364 |
| SHR (95% CI)^‡^ | 1.00 (reference) | 1.06 (1.01-1.12) | 0.011 | 1.05 (0.93-1.19) | 0.434 |
| Vascular dementia |  |  |  |  |  |
| Event, n | 969 | 1,103 |  | 87 |  |
| PYs | 1,090,454 | 1,309,265 |  | 104,710 |  |
| SHR (95% CI)^†^ | 1.00 (reference) | 1.14 (1.03-1.28) | 0.016 | 1.50 (1.19-1.89) | <0.001 |
| SHR (95% CI)^‡^ | 1.00 (reference) | 1.11 (0.99-1.24) | 0.066 | 1.44 (1.14-1.83) | 0.002 |

SHRs (95% CIs) were calculated using the Fine-Gray regression.

^†^Adjusted for age, sex, and body mass index.

^‡^Adjusted for age, sex, body mass index, household income, Charlson comorbidity index, smoking, and moderate-to-vigorous physical activity.

Acronyms: SHR, subdistribution hazard ratio; SLD, steatotic liver disease; MASLD, metabolic dysfunction-associated steatotic liver disease; MetALD, metabolic dysfunction-associated steatotic liver disease with increased alcohol intake; PY, person-year; CI, confidence interval.

**Supplemental Table 4. Standardized mean differences of variables before and after the IPTW.**

| **Variable** | **Non-SLD versus MASLD** | | **Non-SLD versus MetALD** | |
| --- | --- | --- | --- | --- |
|  | **Before the IPTW** | **After the IPTW** | **Before the IPTW** | **After the IPTW** |
| Logit propensity score | 2.6 | 0.0 | 4.1 | -1.0 |
| Age | -0.6 | -0.1 | -1.8 | 2.5 |
| Body mass index | 3.5 | -0.1 | 2.5 | -1.2 |
| Sex | 0.1 | 0.0 | 0.6 | -0.1 |
| Household income | 0.0 | 0.0 | 0.0 | 0.1 |
| Charlson comorbidity index | 0.0 | 0.0 | 0.0 | 0.1 |
| Smoking status | -0.1 | 0.0 | -0.5 | 0.0 |
| MVPA | 0.0 | 0.0 | -0.1 | 0.1 |

Acronyms: IPTW, inverse probability of treatment weighting; SLD, steatotic liver disease; MASLD, metabolic dysfunction-associated steatotic liver disease; MetALD, metabolic dysfunction-associated steatotic liver disease with increased alcohol intake; MVPA, moderate-to-vigorous physical activity.

**Supplemental Table 5. Descriptive characteristics of the participants with non-SLD and MASLD after the inverse probability of treatment weighting.**

| **Variable** | **Non-SLD**  **(n=283,319)** | **MASLD**  **(n=286,134)** |
| --- | --- | --- |
| Age, years | 68.1 (8.4) | 68.0 (7.6) |
| Sex, n (%) |  |  |
| Male | 109,986 (39.1) | 119,890 (41.5) |
| Female | 171,333 (60.9) | 166,244 (58.1) |
| Household income^†^, n (%) |  |  |
| 1^st^ quartile (lowest) | 37,502 (13.3) | 38,266 (13.4) |
| 2^nd^ quartile | 48,971 (17.4) | 49,178 (17.2) |
| 3^4d^ quartile | 66,537 (23.7) | 74,395 (26.0) |
| 4^th^ quartile (highest) | 128,309 (45.6) | 124,296 (43.4) |
| Body mass index, kg/m^2^ | 24.2 (4.8) | 24.1 (4.2) |
| Waist circumference, cm | 80.9 (10.5) | 84.1 (10.4) |
| Systolic blood pressure, mmHg | 127.2 (22.9) | 130.4 (21.9) |
| Diastolic blood pressure, mmHg | 77.0 (14.3) | 78.9 (13.7) |
| Fasting serum glucose, mg/dL | 99.4 (36.7) | 106.7 (41.5) |
| Total cholesterol, mg/dL | 193.0 (54.0) | 204.1 (57.0) |
| HDL-cholesterol, mg/dL | 55.4 (36.7) | 53.5 (39.3) |
| LDL-cholesterol, mg/dL | 117.9 (51.7) | 118.3 (58.4) |
| Triglycerides, mg/dL | 102.2 (71.3) | 170.5 (131.1) |
| Alanine aminotransferase, IU/L | 18.5 (12.6) | 23 (18-32) |
| Aspartate aminotransferase, IU/L | 23.1 (10.7) | 29.8 (28.8) |
| γ-GT, IU/L | 15.8 (9.8) | 34 (24-54) |
| Alcohol consumption, n (%) |  |  |
| No | 230,239 (81.8) | 213,275 (74.5) |
| 1-2 times/week | 36,170 (12.9) | 51,292 (17.9) |
| 3-4 times/week | 9,527 (3.4) | 15,276 (5.3) |
| ≥5 times/week | 5,382 (1.9) | 6,292 (2.2) |
| Cigarette smoking, n (%) |  |  |
| Never | 212,140 (75.4) | 210,042 (73.4) |
| Past | 39,793 (14.2) | 38,871 (13.6) |
| Current | 29,385 (10.5) | 37,222 (13.0) |
| MVPA, n (%) |  |  |
| No | 198,802 (70.7) | 200,729 (70.2) |
| 1–2 times/week | 26,098 (9.3) | 29,398 (10.3) |
| 3–4 times/week | 22,317 (7.9) | 22,649 (7.9) |
| ≥5 times/week | 34,102 (12.1) | 33,358 (11.7) |
| Charlson comorbidity index, n (%) |  |  |
| 0 | 78,026 (27.7) | 80,549 (28.2) |
| 1 | 91,508 (32.5) | 81,982 (28.7) |
| ≥2 | 111,786 (39.7) | 123,603 (43.6) |

Continuous data are presented as mean (standard deviation) and median (interquartile range) if normally distributed and not normally distributed, respectively.

Categorical data are expressed as the number (%).

^†^Proxy for socioeconomic status based on the insurance premium of the National Health Insurance Service.

Acronyms: SLD, steatotic liver disease; MASLD, metabolic dysfunction-associated steatotic liver disease; HDL, high-density lipoprotein; LDL, low-density lipoprotein cholesterol; γ-GT, γ-glutamyl transpeptidase; MVPA, moderate-to-vigorous physical activity.

**Supplemental Table 6. Descriptive characteristics of the participants with non-SLD and MetALD after the inverse probability of treatment weighting.**

| **Variable** | **Non-SLD**  **(n=140,995)** | **MetALD**  **(n=201,374)** |
| --- | --- | --- |
| Age, years | 68.1 (6.0) | 70.6 (28.4) |
| Sex, n (%) |  |  |
| Male | 55,713 (39.5) | 64,747 (32.2) |
| Female | 85,282 (60.5) | 136,627 (67.9) |
| Household income^†^, n (%) |  |  |
| 1^st^ quartile (lowest) | 18,843 (13.4) | 25,069 (12.5) |
| 2^nd^ quartile | 23,759 (16.9) | 47,504 (23.6) |
| 3^4d^ quartile | 34,539 (24.5) | 64,281 (31.9) |
| 4^th^ quartile (highest) | 63,853 (45.3) | 64,520 (32.0) |
| Body mass index, kg/m^2^ | 22.4 (2.5) | 21.2 (11.2) |
| Waist circumference, cm | 78.1 (7.0) | 80.1 (26.3) |
| Systolic blood pressure, mmHg | 126.3 (16.5) | 132.2 (66.6) |
| Diastolic blood pressure, mmHg | 76.6 (10.3) | 79.8 (38.6) |
| Fasting serum glucose, mg/dL | 98.9 (24.3) | 106.7 (101.6) |
| Total cholesterol, mg/dL | 194.8 (38.5) | 201.9 (175.6) |
| HDL-cholesterol, mg/dL | 56.0 (27.9) | 60 (50-73) |
| LDL-cholesterol, mg/dL | 118.4 (37.1) | 99 (66-131) |
| Triglycerides, mg/dL | 106.7 (54.2) | 158 (118-220) |
| Alanine aminotransferase, IU/L | 18.7 (9.3) | 26 (18-34) |
| Aspartate aminotransferase, IU/L | 23.7 (8.1) | 31 (24-40) |
| γ-GT, IU/L | 17.5 (7.8) | 63 (37-129) |
| Alcohol consumption, n (%) |  |  |
| No | 113,767 (80.7) | 0 (0) |
| 1-2 times/week | 18,504 (13.1) | 21,400 (10.6) |
| 3-4 times/week | 5,431 (3.9) | 58,341 (29.0) |
| ≥5 times/week | 3,293 (2.3) | 121,633 (60.4) |
| Cigarette smoking, n (%) |  |  |
| Never | 104,815 (74.3) | 140,742 (69.9) |
| Past | 19,368 (13.7) | 14,886 (7.4) |
| Current | 16,811 (11.9) | 45,746 (22.7) |
| MVPA, n (%) |  |  |
| No | 98,172 (69.6) | 153,727 (76.3) |
| 1–2 times/week | 14,090 (10.0) | 15,894 (7.9) |
| 3–4 times/week | 11,522 (8.2) | 9,429 (4.7) |
| ≥5 times/week | 17,211 (12.2) | 22,324 (11.1) |
| Charlson comorbidity index, n (%) |  |  |
| 0 | 43,987 (31.2) | 73,454 (36.5) |
| 1 | 43,674 (31.0) | 63,338 (31.5) |
| ≥2 | 53,334 (37.8) | 64,582 (32.1) |

Continuous data are presented as mean (standard deviation) and median (interquartile range) if normally distributed and not normally distributed, respectively.

Categorical data are expressed as the number (%).

^†^Proxy for socioeconomic status based on the insurance premium of the National Health Insurance Service.

Acronyms: SLD, steatotic liver disease; MetALD, metabolic dysfunction-associated steatotic liver disease with increased alcohol intake; HDL, high-density lipoprotein; LDL, low-density lipoprotein cholesterol; γ-GT, γ-glutamyl transpeptidase; MVPA, moderate-to-vigorous physical activity.

**Supplemental Table 7. Variance inflation factors for variables used in evaluating subdistribution hazard ratios for incident overall dementia after the inverse probability of treatment weighting across the SLD subtypes.**

| **Variable** | **Variance inflation factor** | |
| --- | --- | --- |
|  | **Non-SLD versus MASLD** | **Non-SLD versus MetALD** |
| Sex | 1.50 | 1.30 |
| Age | 1.04 | 1.23 |
| Body mass index | 1.04 | 1.24 |
| Income | 1.01 | 1.02 |
| CCI | 1.02 | 1.03 |
| Smoking status | 1.48 | 1.30 |
| MVPA | 1.02 | 1.08 |

Acronyms: SLD, steatotic liver disease; MASLD, metabolic dysfunction-associated steatotic liver disease; MetALD, metabolic dysfunction-associated steatotic liver disease with increased alcohol intake; CCI, Charlson comorbidity index; MVPA, moderate-to-vigorous physical activity

**Supplemental Table 8. Subdistribution hazard ratios for incident overall dementia and its subtypes across SLD subtypes after excluding body mass index from the adjustment.**

|  | **No SLD** | **MASLD** | ***P* value** | **MetALD** | ***P* value** |
| --- | --- | --- | --- | --- | --- |
| Overall dementia | 1.00 (reference) | 1.02 (0.99-1.05) | 0.205 | 1.06 (0.96-1.17) | 0.260 |
| Alzheimer’s disease | 1.00 (reference) | 1.01 (0.98-1.05) | 0.489 | 1.03 (0.93-1.14) | 0.550 |
| Vascular dementia | 1.00 (reference) | 1.09 (1.01-1.18) | 0.020 | 1.40 (1.15-1.70) | <0.001 |

Data are subdistribution hazard ratios (95% CIs) using the Fine-Gray regression after adjustment for age, sex, household income, Charlson comorbidity index, smoking, and moderate-to-vigorous physical activity.

Acronyms: SLD, steatotic liver disease; MASLD, metabolic dysfunction-associated steatotic liver disease; MetALD, metabolic dysfunction-associated steatotic liver disease with increased alcohol intake.
